# MoltiTox: a multimodal fusion model for molecular toxicity prediction

**DOI:** 10.3389/ftox.2025.1720651

**Published:** 2025-12-18

**Authors:** Junwoo Park, Sujee Lee

**Affiliations:** 1 Department of Computer Science and Engineering, Sungkyunkwan University, Suwon, Republic of Korea; 2 Department of Systems Management Engineering, Sungkyunkwan University, Suwon, Republic of Korea

**Keywords:** multimodal learning, deep learning, toxicity prediction, Tox21, ^13^C NMR spectra, drug discovery, cheminformatics

## Abstract

**Introduction:**

We introduce MoltiTox, a novel multimodal fusion model for molecular toxicity prediction, designed to overcome the limitations of single-modality approaches in drug discovery.

**Methods:**

MoltiTox integrates four complementary data types: molecular graphs, SMILES strings, 2D images, and ^13^C NMR spectra. The model processes these inputs using four modality-specific encoders, including a GNN, a Transformer, a 2D CNN, and a 1D CNN. These heterogeneous embeddings are fused through an attention-based mechanism, enabling the model to capture complementary structural and chemical information from multiple molecular perspectives.

**Results:**

Evaluated on the Tox21 benchmark across 12 endpoints, MoltiTox achieves a ROC-AUC of 0.831, outperforming all single-modality baselines.

**Discussion:**

These findings highlight that integrating diverse molecular representations enhances both the robustness and generalizability of toxicity prediction models. Beyond predictive performance, the inclusion of ^13^C NMR data offers complementary chemical insights that are not fully captured by structure or language-based representations, suggesting its potential contribution to mechanistic understanding of molecular toxicity. By demonstrating how multimodal integration enriches molecular representations and enhances the interpretability of toxicity mechanisms, MoltiTox provides an extensible framework for developing more reliable models in computational toxicology.

## 1 Introduction

The prediction of chemical toxicity is a critical task in new drug development and safety assessment (Kola and Landis, 2004; Andersen and Krewski, 2009). This process enables the early identification of potentially harmful molecules, minimizing risks to human health and the environment. Traditionally, toxicity evaluation and molecular design processes have relied on biological testing in laboratories, but this approach is fraught with challenges, including high costs and long timelines. Consequently, there has been a significant increase in demand for computational toxicity prediction methods that can replace or supplement these costly experiments (Krewski et al., 2010).

In the field of toxicity prediction, Quantitative Structure-Activity Relationship (QSAR) models have been predominantly used. The QSAR approach predicts toxicity by modeling the mathematical relationship between a molecule’s structure and its biological activity (Sun et al., 2012). It primarily characterizes molecules using numerical representations, such as molecular descriptors (Todeschini and Consonni, 2009). However, QSAR models often fail to capture complex molecular properties, which limits their predictive accuracy. For example, fingerprint-based QSAR models are known to struggle with “activity cliffs,” a phenomenon where minor structural changes cause significant shifts in biological activity (Cruz-Monteagudo et al., 2014).

With the recent rapid advancements in artificial intelligence and deep learning, a new paradigm has emerged in chemical and biological data analysis (Chen et al., 2018; Min et al., 2017). Various models have been proposed utilizing different molecular representations, including graphs, Simplified Molecular Input Line Entry System (SMILES) strings (Weininger, 1988), and 2D images. Graph-based models, such as Graph Neural Networks (GNNs), can directly learn the molecular graph structure, including atomic properties, bond types, and inter-atomic graph distances, thereby efficiently capturing molecular characteristics (Kearnes et al., 2016). Indeed, GNNs have achieved excellent performance in toxicity prediction and are widely used in the field of chemistry (Jiang et al., 2021). In parallel, Transformer-based Chemical Language Models that leverage SMILES strings have also gained attention (Chithrananda et al., 2020). SMILES strings contain a molecule’s linear structure and chemical syntax information. By analyzing these strings with the Transformer architecture, it is possible to more effectively capture complex chemical properties and structural patterns of molecules (Ross et al., 2022). Furthermore, approaches that represent molecules as 2D images for analysis with Convolutional Neural Network (CNN) based models have also been developed. These image-based methods have demonstrated the ability to automatically learn structurally significant features closely related to toxicity, such as functional groups and aromatic rings (Goh et al., 2017b; Zeng et al., 2022).

Deep learning approaches that utilize these diverse representations have demonstrated performance surpassing that of traditional QSAR models on standard toxicity prediction benchmark datasets (Wu et al., 2018), suggesting their capability to effectively model the complex and multidimensional mechanisms of toxicity. However, most existing toxicity prediction models utilize a maximum of only two or three representation methods (Manolache et al., 2024; Rollins et al., 2024). Therefore, this study aims to enhance toxicity prediction performance through a more comprehensive fusion of various molecular representation methods.

In this paper, we propose MoltiTox, a multimodal fusion model for toxicity prediction. To construct a holistic molecular representation, the model integrates four complementary data types: molecular graphs, SMILES strings, 2D images, and ^13^C NMR spectra. The first three are foundational computational representations, while the fourth provides unique spectroscopic information that complements structural features. By fusing this complementary information, we aim to overcome the limitations of single-representation models. For instance, while graph-based models effectively capture the topological features of a molecule (Gilmer et al., 2017), structural relationships derived from bond order and chemical syntax within the molecule can be better reflected by SMILES-based models (Ross et al., 2022). Similarly, image-based models recognize 2D structural patterns, such as motifs and functional groups associated with toxicity (Zeng et al., 2022). Including NMR data provides complementary chemical insights, as spectral shifts encode information about the electronic environment and reactivity of functional groups that may not be fully captured by structural representations (Kim et al., 2023).

**FIGURE 1:**
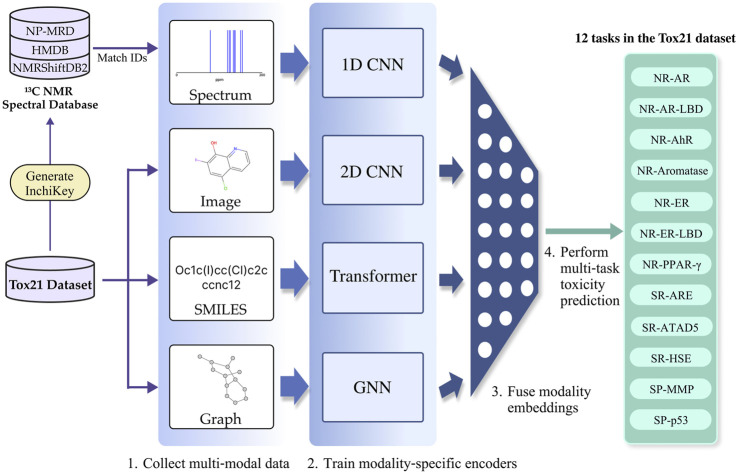
MoltiTox pipeline overview. The workflow illustrates multimodal molecular processing: (1) extraction of four data types, ^13^C NMR spectrum (nuclear magnetic resonance), 2D image, SMILES string (Simplified Molecular Input Line Entry System), and molecular graph, from each compound (example: ‘Oc1c(I)cc(Cl)c2cccnc12’); (2) encoding of each modality via GNN (Graph Neural Network), Transformer, 2D CNN (Convolutional Neural Network), and 1D CNN to obtain embeddings; (3) fusion of embeddings into an integrated molecular representation; and (4) multi-task toxicity prediction across 12 Tox21 endpoints.

Notably, the ^13^ C NMR spectrum utilized in this study is a representation rarely employed in toxicity prediction research, offering unique information about the chemical environment of a molecule. The peaks in a^13^ C NMR spectrum reflect the electronic and orbital environments of carbon atoms within a molecule. This information is closely linked to the compound’s chemical reactivity and physical properties, suggesting its potential to contain valuable data for toxicity prediction (Verma and Hansch, 2011). MoltiTox aims to combine all four of these representations to capture a more holistic view of molecular structure and properties, thereby performing more accurate and robust toxicity predictions. The overall pipeline of the MoltiTox are summarized in Figure 1.

To ensure comparability of results and reproducibility as a standard benchmark, this study was evaluated using the widely adopted Tox21 dataset for toxicity prediction. Tox21 is an outcome of the ‘Toxicology in the 21st Century’ program, a collaborative initiative led by U.S. government agencies including NIH, EPA, and FDA. Released to the public for the 2014 Tox21 Challenge (Tox21 Challenge, 2014), it covers approximately 10,000 chemical compounds. The dataset includes binary labels for a total of 12 *in vitro* toxicity endpoints for each compound, comprising seven nuclear receptor (NR) tasks and five stress response (SR) tasks. The deep learning-based pipeline DeepTox (Mayr et al., 2016) achieved the highest performance in the original Tox21 Challenge. Subsequently, models applying state-of-the-art representation techniques, such as GNNs and Transformer-based chemical language models, have demonstrated superior performance on this toxicity prediction task.

Experimental results show that the proposed model, MoltiTox, outperforms all single-modality baseline models, demonstrating that the representation-level fusion of complementary modalities yields superior predictive performance. Notably, the inclusion of ^13^ C NMR spectra as an input significantly improved performance on certain tasks. This result suggests an enhancement in prediction accuracy for toxicity endpoints where electronic and metabolic characteristics are critical determining factors.

Overall, this study contributes to the field of molecular toxicity prediction in three key ways. First, it introduces a unified multimodal framework that integrates four complementary molecular representations: molecular graphs, SMILES strings, 2D images, and NMR spectra, providing a more comprehensive foundation for toxicity modeling. Second, it incorporates ^13^ C NMR data as a novel and informative modality, offering unique spectroscopic features that enrich molecular representation. Third, it demonstrates that multimodal fusion enhances robustness and generalizability across diverse toxicity endpoints in the Tox21 benchmark.

The remainder of this paper is organized as follows. Section 2 reviews related research on molecular representation and multimodal learning in chemistry. Section 3 describes the dataset, preprocessing procedures, and model architecture. Section 4 presents the experimental design and evaluation protocol, followed by results and analysis in Section 5. Section 6 discusses the implications of the findings, and Section 7 outlines the limitations and future directions. Finally, Section 8 concludes the paper.

## 2 Related work

### 2.1 Graph-based models

Graph Neural Networks (GNNs) were first proposed as a framework for directly processing graph-structured data (Scarselli et al., 2008). In the context of molecular applications, atoms can be represented as nodes and bonds as edges (Duvenaud et al., 2015). GNNs are typically implemented as a message-passing framework, in which each node iteratively updates its state by receiving messages from its neighboring nodes (Gilmer et al., 2017). Through this process, both local structural information and global topological information of the molecular graph can be captured. Various GNN architectures have been proposed for predicting molecular properties, with notable examples including Graph Convolutional Networks (GCN) (Kipf and Welling, 2016), Graph Sample and Aggregate (GraphSAGE) (Hamilton et al., 2017), and Graph Attention Networks (GAT) (Veličković et al., 2017). Among these, the Graph Isomorphism Network (GIN) proposed by Xu et al. (2018) is particularly noteworthy. GIN employs a sum-based aggregation function to combine neighboring node features, followed by transformation through a multi-layer perceptron (MLP). It has been theoretically proven to possess expressive power equivalent to the Weisfeiler-Lehman (WL) graph isomorphism test (Weisfeiler and Leman, 1968) in distinguishing graph structures. Based on these strengths, this study adopts GIN to construct the graph encoder for processing molecular graphs. However, in practice, GNNs can over-squash long-range interaction information during the message-passing process, potentially losing important information (Alon and Yahav, 2020).

### 2.2 SMILES-based models

Another widely used method for representing molecules is the SMILES string (Weininger, 1988). SMILES represents a chemical structure as a linear string, enabling the application of NLP techniques to problems in chemistry (Honda et al., 2019). While early research modeled SMILES using Recurrent Neural Networks (RNNs) or one-hot encoding (Goh et al., 2017a), Transformer-based chemical language models have recently shown superior performance (Schwaller et al., 2019). MolFormer-XL (Ross et al., 2022) is a Transformer-based model trained in a self-supervised manner on approximately 1.1 billion SMILES strings. This model treats SMILES as a “chemical language” and uses an attention mechanism to simultaneously learn both the substructures within a molecule and the spatial interatomic distances, thereby effectively capturing both the chemical and spatial properties of molecules. As a result, it has shown excellent performance when fine-tuned for downstream tasks such as toxicity prediction. In this study, we have adopted MolFormer-XL as the constituent model for the SMILES encoder, due to its ability to effectively learn the sequential properties of molecules. However, SMILES-based chemical input models convert the ring structures of molecules into linear strings by breaking the rings and using numbers or parentheses to indicate connectivity. During this process, atoms that are structurally close in the actual molecule may appear far apart in the string, which can prevent certain local structures or bonding patterns from being adequately captured (Lee and Nam, 2022).

### 2.3 Image-based models

Converting molecules into 2D structural images for input into CNN-based models is one of the approaches for molecular property prediction. Image-based feature learning using molecular images effectively complements visual patterns that are difficult for conventional fingerprint methods to capture. In this study, a ResNet-based architecture from ImageMol (Zeng et al., 2022) was adopted as the model for processing image inputs. ImageMol pre-trained a ResNet-18 model in a self-supervised manner on approximately 10 million unlabeled 2D molecular images extracted from the PubChem database, using pre-training tasks such as image masking, context prediction, and structural jigsaw puzzles. The resulting features achieved competitive or superior performance to text- and graph-based models across various molecular property prediction benchmarks. The pre-trained model effectively captures functional groups such as = O, –OH, and–NH_2_, as well as structural motifs like benzene rings, from 2D images. Furthermore, analysis using Grad-CAM (Selvaraju et al., 2017) confirmed that the model also attends to the overall shape of the molecule (Zeng et al., 2022).

### 2.4 Spectrum-based models

Experimentally obtained spectral data provide useful information about molecular structure from a different perspective than conventional structure-based representations. In particular, the ^13^C NMR spectrum sensitively reflects the chemical environment of carbon atoms within a molecule, allowing it to be used as an important molecular descriptor (Verma and Hansch, 2011). The unique chemical environment of each carbon atom forms a peak at a specific chemical shift, and the entire NMR spectrum, composed of a combination of these peaks, can be considered a molecular fingerprint. Although there have been some attempts to utilize NMR spectra in molecular analysis, such as DeepSAT (Kim et al., 2023) for structure prediction, research integrating NMR spectra into toxicity prediction models remains rare. Yang et al. (2021) developed CReSS (Cross-modal Retrieval between Spectrum and Structure), which demonstrated that spectra and molecules can be interconnected with high accuracy by mapping ^13^C NMR spectra and molecular structures into the same embedding space through deep contrastive learning. We employ the pre-trained NMR encoder from CReSS to extract fixed-length embeddings from the ^13^C NMR spectrum of each molecule.

### 2.5 Multimodal approaches

Over the past few years, there has been a growing number of attempts to improve the performance of molecular property prediction by combining various data modalities. Although some studies on multimodal deep learning for molecular data existed prior to this research, they generally combined only two or three modalities (Rollins et al., 2024; Wang et al., 2025; Manolache et al., 2024). Manolache et al. (2024) integrates SMILES, 2D graphs, and 3D conformers by concatenating modality-specific embeddings into a unified sequence processed through a downstream transformer. Rollins et al. (2024) fuses language representations from ChemBERTa-2 with graph representations by mapping SMILES tokens to graph nodes and concatenating their features. Wang et al. (2025) developed an asymmetric contrastive learning framework that pairs molecular graphs with multiple chemical modalities including SMILES, images, and spectroscopic data through pairwise contrastive loss.

Recent advances have also explored large language model-based multimodal fusion. Boyar et al. (2025) proposed LLM-Fusion for materials property prediction, integrating SMILES, SELFIES (SELF-referencing Embedded Strings), molecular fingerprints, and text descriptions through LLM self-attention mechanisms. Li et al. (2025) introduced ChemVLM, a multimodal large language model that combines visual information (molecular structures, reaction diagrams) with textual data using a Vision Transformer (ViT)-Multi-Layer Perceptron (MLP)-LLM architecture. Earlier work by Karim et al. (2019) proposed a multimodal model for toxicity prediction by transforming molecules into three distinct representations: SMILES strings, 2D molecular images, and descriptor-based physicochemical numerical features. The outputs from their respective Recurrent Neural Network (RNN), CNN, and Fully Connected Neural Network (FCNN) models were subsequently integrated through an ensemble approach. The results showed that the ensemble method, which combined diverse inputs, achieved higher performance than single-modality models, confirming the potential of multimodal learning. This suggests that different data representations provide complementary signals, and combining multiple representations can lead to more accurate predictions.

Therefore, MoltiTox fuses graphs, SMILES, images, and spectral data within a unified deep learning architecture, inputting each into GNN, Transformer, 2D CNN, and 1D CNN-based encoders, respectively. Building on this foundation, the next section presents MoltiTox’s materials and methodology, including dataset description, processing methods for each modality, and the fusion network architecture.

## 3 Materials and methods

This section describes the acquisition and preprocessing of the four modalities from the Tox21 dataset. It then details the architecture of the modality-specific encoders and the overall structure of the MoltiTox model that integrates them.

### 3.1 Dataset

For molecular toxicity prediction, this study utilizes the Tox21 benchmark dataset provided by MoleculeNet (Wu et al., 2018). The Tox21 dataset consists of 7,831 unique chemical compounds and 12 binary toxicity endpoints. Each compound-endpoint pair is labeled as active (1), inactive (0), or missing. The 12 endpoints are classified into two groups: seven nuclear receptor (NR) panels (NR–AR, NR–AhR, NR–AR–LBD, NR–ER, NR–ER–LBD, NR–Aromatase, and NR–PPAR– γ ) and five stress response (SR) panels (SR–ARE, SR–ATAD5, SR–HSE, SR–MMP, and SR–p53).

Each compound is provided in the form of a SMILES (Weininger, 1988) string. We selected 7,823 valid molecules that could be successfully converted into molecular graphs and images using the MolFromSmiles function from RDKit (RDKit, 2006). These serve as the inputs for the Graph, SMILES, and Image encoders, respectively. To acquire the spectrum data, which is not directly provided in the Tox21 dataset, we first converted the SMILES strings of the compounds to International Chemical Identifier Keys (InChIKeys) using RDKit. We then matched these InChIKeys against three public spectral databases in sequential order: NMRShiftDB (Steinbeck et al., 2003), NP-MRD (Wishart et al., 2022), and HMDB (Wishart et al., 2007). Once a spectrum was identified in one database, subsequent databases were not queried for that compound, thereby preventing duplicates. As a result, we were able to obtain valid ^13^ C NMR spectra for a total of 2,851 compounds, which were used as input for the Spectrum encoder.

Following the procedures outlined above, we constructed the input data for each of the four encoders in MoltiTox. Among these, the molecular graphs were dynamically generated from SMILES strings via RDKit during the model training process. The 2D images were pre-rendered from each SMILES string into images using RDKit and saved for use in training. For the spectral data, the ^13^C NMR spectra were transformed into vectors and saved for training.

### 3.2 Architecture

**FIGURE 2:**
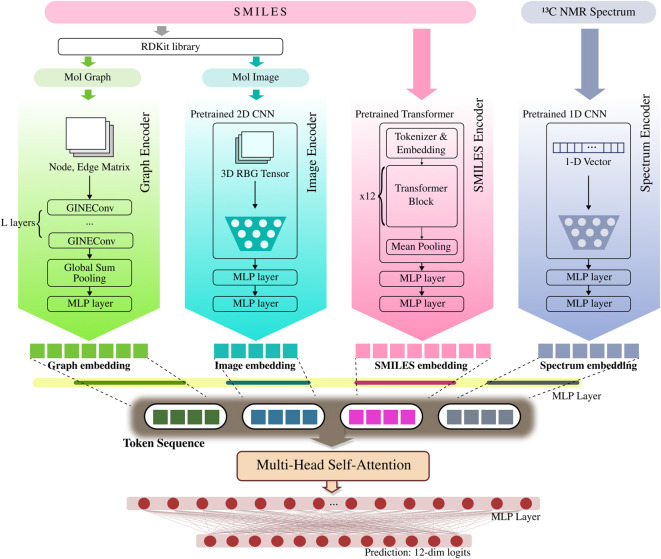
MoltiTox architecture. Overview of the MoltiTox pipeline. Four modality-specific encoders process molecular inputs: (1) a GINEConv (Graph Isomorphism Network with Edge features Convolution) for graphs, (2) a pretrained 2D CNN (ResNet-18 backbone) for molecular images, (3) a pretrained 12-layer Transformer for SMILES strings, and (4) a pretrained 1D CNN for ^13^C NMR spectra. All embeddings are projected into a shared latent space and fused via multi-head self-attention. The fused representation passes through an MLP to predict 12 toxicity endpoints.

To fully leverage the complementary strengths of diverse molecular representations and enhance overall predictive performance, this study designs a four-branch architecture where each encoder specializes in a different modality. By extracting respective features from graphs, SMILES, images, and spectral data, the model constructs a robust embedding space. Since the outputs of the encoders have different dimensions, they are passed through individual MLP projection layers to be mapped to a uniform embedding dimension before fusion. Finally, these normalized vectors are fed into a fusion network to be combined, and the resulting vector is input to a classifier for the final toxicity prediction. The overall architecture of the model, presented in Figure 2, consists of four parallel encoders whose outputs are subsequently combined.

#### 3.2.1 GNN-based graph branch

The Graph encoder is implemented based on the Graph Isomorphism Network (GIN) (Xu et al., 2018), a GNN model known for its high expressive power. It processes molecular graphs where atoms are represented as nodes and bonds as edges. Each atom node is represented as a 78-dimensional vector, which combines one-hot encodings for categorical features (e.g., atomic symbol, hybridization) and scalar values for others (e.g., formal charge, aromaticity). That is, for a molecule with N atoms, a node feature matrix $X\in R^{N\times 78}$ is formed. Furthermore, we adopt an edge-aware variant of GIN (GINEConv) (Hu et al., 2020). The B undirected bonds in the molecule are converted into 2B directed edges (one “forward” and one “backward” for each bond) to enable bidirectional message passing. This yields an edge index matrix $E\in Z^{2\times 2B}$ , which records the source and destination node indices for each edge, and an edge feature matrix $A\in R^{2B\times 4}$ , composed of 4-dimensional one-hot encoded vectors indicating the bond type (single, double, triple, or aromatic). Finally, these three matrices, X , E , and A , are passed to GINEConv layers for learning.

Specifically, the GIN is constructed by stacking L GINEConv layers (Xu et al., 2018; Hu et al., 2020), each composed of a two-layer MLP with a hidden dimension h . In each GINEConv layer, the representation of a node v , denoted as $h_{v}^{\left(k\right)}$ , is updated as follows:

$$h_{v}^{\left(k\right)}={MLP}^{\left(k\right)}\left(\left(1+\varepsilon^{\left(k\right)}\right)\;h_{v}^{\left(k-1\right)}+\underset{u\in N\left(v\right)}{\sum }ReLU\left(h_{u}^{\left(k-1\right)}+e_{u,v}\right)\right),$$

where $h_{v}^{\left(k\right)}\in R^{h}$ is the feature vector of node v at the k -th layer, $\varepsilon^{\left(k\right)}$ is a learnable scalar parameter for each layer, N(v) is the set of neighboring nodes of node v , $e_{u,v}\in R^{4}$ is the feature vector of the edge (u,v) , ${MLP}^{\left(k\right)}$ is a two-layer multi-layer perceptron, and ReLU(⋅) is the non-linear activation function. After the node representations are updated by the MLP in each layer, batch normalization, ReLU activation, and dropout are sequentially applied. Following L rounds of message passing, the node embeddings are aggregated per graph using global sum pooling. The resulting h -dimensional pooled vector then passes through an MLP projection head with ReLU activation and dropout, producing the final graph embedding $h_{\text{graph}}\in R^{d_{g}}$ .

#### 3.2.2 Transformer-based SMILES branch

The SMILES encoder utilizes MolFormer-XL (Ross et al., 2022), a Transformer model pre-trained on a large-scale dataset of molecular SMILES. As proposed in Ross et al. (2022), each SMILES string is padded and truncated to a maximum length of 202 tokens. From this, token IDs $s_{\text{id}}\in Z^{202}$ and an attention mask $s_{\text{mask}}\in {\left\{0,1\right\}}^{202}$ are generated and fed into the Attention layers within the Transformer. The Transformer internally processes these inputs through operations such as linear attention, add & norm, and feed forward to generate token embeddings of dimension H . These token embeddings are then summarized into a single vector via mean pooling over the entire sequence. This vector is refined through a two-layer MLP projection head: the first stage applies a linear transformation from dimension h to h , followed by dropout and GELU activation; the second stage repeats these operations with a linear transformation from dimension h to $d_{s}$ , ultimately yielding a $d_{s}$ -dimensional SMILES embedding $h_{\text{smiles}}\in R^{d_{s}}$ . This design follows the Tox21 fine-tuning methodology proposed in Ross et al. (2022).

#### 3.2.3 2D CNN-based image branch

The Image encoder employs the ResNet (He et al., 2016) architecture from ImageMol (Zeng et al., 2022), a molecular image model pre-trained on a large-scale dataset of molecular images. During training, the image file corresponding to each molecule undergoes data augmentation, including resizing, random horizontal flipping, rotation, and random grayscaling. It is then normalized using the standard ImageNet (Deng et al., 2009) mean of [0.485,0.456,0.406] and standard deviation of [0.229,0.224,0.225] . The transformed image $I\in R^{3\times 224\times 224}$ (RGB channels) is passed through the ResNet backbone without its final fully connected layer, producing a 512-dimensional feature vector. This feature vector is then refined by a two-layer MLP projection head, which uses ReLU activation and dropout, to produce the final $d_{i}$ -dimensional image embedding $h_{\text{image}}\in R^{d_{i}}$ .

#### 3.2.4 1D CNN-based spectrum branch

The Spectrum encoder employs the NMR encoder from CReSS (Yang et al., 2021), pre-trained on a large dataset of molecular ^13^C NMR spectra. Following the preprocessing procedure described in Yang et al. (2021), each spectrum was discretized and converted into a binary representation of spectral peaks. The vector is processed by the pre-trained model to extract a 768-dimensional feature vector, which is refined by a two-layer MLP projection head using ReLU activation and dropout to generate the final $d_{sp}$ -dimensional spectrum embedding $h_{\text{spectrum}}\in R^{d_{sp}}$ .

#### 3.2.5 Multimodal fusion and prediction

The modality-specific embeddings $h_{\text{graph}},h_{\text{smiles}},h_{\text{image}},h_{\text{spectrum}}$ are first linearly projected to a common dimension $d_{e}$ , followed by a ReLU activation:

$$g=ReLU\left(W_{g}h_{\text{graph}}\right),\;s=ReLU\left(W_{s}h_{\text{smiles}}\right),\;i=ReLU\left(W_{i}h_{\text{image}}\right),\;sp=ReLU\left(W_{sp}h_{\text{spectrum}}\right),$$

where $g,s,i,sp\in R^{d_{e}}$ are the projected embeddings, and $W_{g},W_{s},W_{i},W_{sp}$ are learnable projection matrices.

$$X=\left[g;s;i;sp\right]\in R^{4\times d_{e}},$$

which is then passed through a shared multi-head self-attention module (Vaswani et al., 2017) to obtain

$$Z=\text{MultiHead}\left(X,X,X\right)\in R^{4\times d_{e}},$$

where $Z_{m,:}$ denotes the *m*-th row of Z . The attention outputs are mean-pooled to form the fused representation,

$$fused=\frac{1}{4}\overset{4}{\underset{m=1}{\sum }}Z_{m,:}\in R^{d_{e}}.$$

Finally, the fused embedding is processed through a Linear → ReLU → Dropout block and then passed to a final MLP head, which produces the output vector

$$y\in R^{n_{\text{tasks}}}.$$

As the Tox21 dataset comprises 12 endpoints, $n_{\text{tasks}}$ is 12.

## 4 Experiments

This section details the experimental design and evaluation protocols used in our study. We first describe the scaffold-based data partitioning strategy implemented for model validation, followed by an overview of the baseline models used for performance comparison. Subsequently, we outline the training procedures for the modality-specific encoders and the multimodal model. The section concludes with the final evaluation protocol, which was designed to ensure the reproducibility and robustness of our findings.

### 4.1 Data splitting and partitioning

To rigorously evaluate model generalization, we partitioned the compound dataset using the scaffold split technique (Bemis and Murcko, 1996). This method avoids the overestimation of predictive power common in random splitting, where structurally similar compounds can appear in both training and test sets. The scaffold-based approach groups molecules with shared core structures into a single data partition (Wu et al., 2018). This strategy better simulates real-world drug discovery scenarios by testing a model’s ability to predict properties for entirely new chemical classes.

We generated Bemis-Murcko scaffolds for all 7,823 compounds using the RDKit library. Molecules were then grouped by their common scaffold, and these groups were allocated to training, validation, and test sets in an 8:1:1 ratio. This process guarantees no scaffold overlap between partitions, which is critical for preventing information leakage and fairly assessing performance on unseen chemical structures. To ensure reliable evaluation, we repeated the data partitioning five times using different random seeds. Our MoltiTox model was then trained and evaluated independently on each of these five splits. Unlabeled endpoints were assigned a value of -1 and masked during the training process to manage missing data.

The ^13^C NMR spectral data were available for only 2,851 compounds. The Spectrum encoder was trained exclusively on this NMR-available subset to learn meaningful spectral representations. However, to enable fair comparison and avoid data selection bias, MoltiTox was trained and evaluated on the full dataset (7,823 compounds) by introducing a learnable missing token.

### 4.2 Model training

We trained each modality-specific encoder (Graph, SMILES, Image, and Spectrum) individually to serve as a fixed feature extractor during the multimodal fusion stage. For each encoder, we used Optuna (Akiba et al., 2019), which optimizes hyperparameters using the Tree-structured Parzen Estimator (TPE) algorithm (Bergstra et al., 2011). This method models the relationship between hyperparameter configurations and validation performance through nonparametric density estimation, adaptively sampling promising configurations while maintaining sufficient exploration diversity. All models were trained for a maximum of 50 epochs with a batch size of 32, using early stopping (patience = 10) based on the validation ROC-AUC score. The SMILES and Image encoders were fine-tuned end-to-end with their pre-trained models (Ross et al., 2022; Zeng et al., 2022). Conversely, to prevent overfitting with the smaller spectrum dataset, we froze the pre-trained 1D CNN in the spectrum encoder and trained only its MLP projection head. Furthermore, each modality-specific encoder was evaluated on the test set to serve as a baseline for comparison with MoltiTox.

When training the multimodal fusion network, a learnable missing token was introduced to represent compounds without available NMR spectra. This trainable parameter was initialized to zeros with the same dimension as the spectral embedding. When NMR data were unavailable, the model used the learnable missing token as a substitute for the spectral embedding. During fusion training, all fine-tuned encoders were kept frozen, while only the fusion network and the missing token parameters were updated. This design allowed the model to learn an appropriate representation for the missing spectrum state while preserving the fine-tuned encoder weights. Hyperparameter optimization for the fusion network (e.g., embedding dimension, hidden dimension, dropout rate) was conducted in the same manner as described for the single-modality encoders, with a maximum of 30 epochs and early stopping (patience = 5). The optimal hyperparameters, corresponding to the median validation performance across five independent runs, are summarized in the Supplementary Material.

After training all modality-specific encoders and optimizing the multimodal fusion network, the final models were configured with the optimal hyperparameters identified during the search. Each model was then trained for the number of epochs determined by the early stopping point in its corresponding validation run. We evaluated performance by calculating the ROC-AUC for each of the 12 toxicity endpoints on the fixed test sets. The final metrics were obtained by averaging the results across five independent scaffold splits. In Section 5, we report the mean and standard deviation of ROC-AUC scores for each task, along with the macro-average across all endpoints.

### 4.3 Benchmarking models

To comprehensively evaluate the performance of MoltiTox, we selected a diverse set of state-of-the-art models as benchmarks. These models represent leading approaches across different data modalities and learning paradigms in molecular representation learning. To ensure a fair comparison, all benchmark models were trained and evaluated using the identical data splits employed for our experiments.

For benchmarking, we first included an SVM classifier (Cortes and Vapnik, 1995) as a classical machine learning baseline. Unlike the deep learning models that leverage graphs, sequences, or images, the SVM operates on fixed-length numerical feature vectors. Specifically, we used Extended-Connectivity Fingerprints (ECFP) (Rogers and Hahn, 2010), a widely adopted molecular descriptor in cheminformatics. ECFP encodes molecular structures as binary vectors by capturing circular atomic neighborhoods at a specified radius. We generated ECFP4 fingerprints (radius = 2) with 1024 bits using RDKit (RDKit, 2006). The SVM was trained and implemented using scikit-learn (Pedregosa et al., 2011) with a radial basis function (RBF) kernel. This traditional machine learning approach serves as a fundamental baseline, as SVMs with ECFP have been frequently used in molecular property prediction studies (Wu et al., 2018).

For graph-based representations, we included three powerful models. D-MPNN (Yang et al., 2019) was chosen to represent a highly effective supervised GNN architecture. MolCLR (Wang et al., 2022) was selected as a leading example of self-supervised learning via a contrastive framework. To represent the state-of-the-art in large-scale predictive pre-training, we included GROVER-large (Rong et al., 2020), a graph transformer that integrates message passing with global self-attention.

To benchmark against SMILES sequence and image-based approaches, we included MolFormer-XL (Ross et al., 2022) and ImageMol (Zeng et al., 2022). The specific architectures of these two models, which also serve as the foundation for the SMILES and Image encoders in MoltiTox, are detailed in Section 2. For MolFormer-XL, the original model architecture already incorporates a two-layer MLP projection head, which we retained in the SMILES encoder. In contrast, for ImageMol, we evaluated the model in its original form without any additional projection layers to ensure a fair comparison with existing literature. This architectural difference explains the performance discrepancy between the ImageMol benchmark and the Image encoder, which includes an additional two-layer MLP projection head for improved feature extraction.

## 5 Results

We thoroughly evaluated the predictive performance of our multimodal model, MoltiTox, against its single-modality components on the Tox21 test set. To ensure robust and reproducible findings, our evaluation protocol averaged the performance across five independent runs on distinct scaffold splits (Wu et al., 2018). This section presents an analysis of the overall performance, task-specific contributions, and a comparison to state-of-the-art models.

### 5.1 Overall performance

The aggregate performance metrics in Table 1 demonstrate the synergistic benefit of multimodal fusion.

**TABLE 1:** Mean ( ± std) ROC-AUC scores and computation times for train, valid, and test sets.

Model (encoder)	ROC-AUC			Computation time (s)	
	Train	Valid	Test (p-value)	Training	Inference
Single-modality baselines					
Graph	0.923±0.015	0.799±0.027	0.805±0.027 (0.004)	114.25±23.85	0.39
SMILES	0.909±0.048	0.810±0.027	0.811±0.041 (0.050)	65.74±11.93	0.54
Image	0.837±0.027	0.744±0.033	0.723±0.043 (0.005)	117.24±13.49	0.55
Spectrum	0.854±0.076	0.710±0.056	0.679±0.053 (0.002)	16.88±12.65	0.04
Two-modality baselines					
Graph + SMILES	0.989±0.006	0.801±0.023	0.825±0.027 (0.081)	11.37±7.78	0.97
Graph + image	0.954±0.016	0.811±0.021	0.819±0.026 (0.012)	80.79±35.98	0.97
Graph + spectrum	0.934±0.018	0.798±0.021	0.815±0.027 (0.032)	26.02±14.19	0.48
SMILES + image	0.990±0.009	0.803±0.022	0.813±0.026 (0.003)	33.06±20.10	1.12
SMILES + spectrum	0.986±0.013	0.793±0.012	0.803±0.025 (0.004)	19.92±13.71	0.61
Image + spectrum	0.900±0.023	0.766±0.025	0.758±0.026 (0.000)	70.28±73.57	0.58
Multimodal models					
MoltiTox-3	0.989±0.007	0.790±0.018	0.824±0.026 (0.054)	27.79±12.87	1.56
MoltiTox	0.992±0.004	0.795±0.023	0.831±0.023 (−)	55.44±29.48	1.69

The ROC-AUC values represent the result over five independent scaffold splits. P-values are computed using paired t-tests comparing each model with MoltiTox. Computation time (in seconds) indicates the total training time and inference time per test run.

Bold values indicate the highest test ROC-AUC across all models.

The complete four-modality model, MoltiTox, achieved the highest overall performance, outperforming all single-modality and two-modality baselines. Among single-modality models, the SMILES encoder achieved the best performance with a mean ROC-AUC of 0.811±0.041 , followed by the Graph encoder (0.805±0.027) , Image encoder (0.723±0.043) , and Spectrum encoder (0.679±0.053) . Among two-modality combinations, Graph + SMILES achieved the highest performance (0.825±0.027) , closely followed by Graph + Image (0.819±0.026) and Graph + Spectrum (0.815±0.027) . Notably, MoltiTox-3 (0.824±0.026) , which includes Graph + SMILES + Image, performed comparably to the best two-modality baseline. MoltiTox (0.831±0.023) surpassed all baselines, demonstrating the added value of comprehensive four-modality fusion. These findings support our central hypothesis: integrating complementary information from heterogeneous sources yields a more comprehensive molecular representation than any single modality can provide alone. Furthermore, the standard deviation across the five runs offers insight into model stability. MoltiTox exhibited the lowest variance (±0.023) among all tested configurations. This suggests that multimodal fusion acts as an implicit regularizer; by leveraging diverse feature spaces, the model reduces its sensitivity to specific data partitions and achieves more consistent predictions (Cronin et al., 2025).

In addition to test performance, training and validation ROC-AUC scores provide insight into the models’ generalization behavior. Single-modality models such as Graph and SMILES showed moderate train–valid gaps (approximately 0.12 and 0.10, respectively), indicating limited overfitting and stable learning. By contrast, multimodal models achieved higher training scores (often above 0.98) but maintained comparable validation performance, suggesting that the additional modalities increased representational capacity without causing severe overfitting. MoltiTox and MoltiTox-3 exhibited balanced train–valid trends, supporting that their performance gains on the test set arise from improved feature integration rather than memorization of training data.

Notably, the inclusion of ^13^C NMR spectral data provided a net positive contribution. Despite being available for only 36% of the compounds, its integration elevated the mean ROC-AUC from 0.824 to 0.831. Additionally, it reduced prediction variance from ±0.026 to ±0.023 , indicating that spectral information not only improves accuracy but also enhances model robustness. This result indicates that the unique structural and electronic information in NMR spectra is highly informative for certain toxicity endpoints, outweighing the limitations of data sparsity.

Beyond predictive performance, Table 1 also reports total computation time to evaluate model efficiency. Since all modality-specific encoders were fixed during fusion, the reported training time for MoltiTox and MoltiTox-3 reflects only the optimization of the fusion network, not the total time to train all encoders. Single-modality models such as the Graph and Image encoders required the longest total training durations (over 110 s on average), largely due to their deeper architectures and end-to-end optimization. In contrast, multimodal fusion models like MoltiTox-3 and MoltiTox completed training in 28 s and 55 s, respectively, demonstrating that integrating multiple pretrained encoders can be computationally efficient while maintaining superior predictive performance. Inference time differences across models remained relatively small (under 2 s), indicating that multimodal fusion preserves practical deployability.

A comprehensive summary of additional evaluation metrics, including Accuracy, Balanced Accuracy, Cohen’s Kappa, Matthews Correlation Coefficient (MCC), Sensitivity, and Specificity for all models across train, validation, and test splits, is provided in the Supplementary Material. These results further support the stability of the multimodal fusion approach beyond ROC-AUC performance.

### 5.2 Ablation analysis

To further examine whether the attention-based fusion captures complementary rather than redundant information across modalities, we conducted a comprehensive ablation study involving single-, dual-, and multimodal (three- and four-modality) configurations (Table 1). Performance consistently improved with the addition of modalities, confirming that the fusion network effectively integrates heterogeneous representations.

Among the dual-modality settings, the Graph–SMILES combination achieved the strongest performance (0.825±0.027) , confirming that topological graph features and sequential chemical language features form a robust backbone for molecular representation. The Graph–Image combination also performed competitively (0.819±0.026) , suggesting that 2D structural visual patterns provide auxiliary spatial cues to graph-derived topologies. Although the Spectrum encoder alone performed modestly (0.679±0.053) , it improved predictive accuracy when paired with other modalities, especially Graph (0.815±0.027) , indicating that ^13^C NMR spectra provide complementary information on electronic and structural environments that are not captured by conventional graph or sequence representations.

The three-modality backbone (MoltiTox-3: Graph + SMILES + Image) already exhibited robust and well-balanced performance (0.824±0.026) , and the addition of the ^13^C NMR modality further improved the mean ROC-AUC to 0.831±0.023 . To verify whether this improvement was statistically reliable, we performed paired two-tailed t-tests across the five scaffold splits, comparing each model configuration against the full four-modality MoltiTox. The four-modality model achieved a higher mean ROC-AUC than MoltiTox-3 (p=0.054) , indicating a marginally significant and consistent improvement trend across all splits. Furthermore, MoltiTox significantly outperformed all single-modality baselines and dual-modality configurations (e.g., Graph + Spectrum, SMILES + Image) with p≤0.05 , demonstrating its overall robustness. Given that only 36% of compounds contain spectral data, this improvement underscores that NMR-derived features capture mechanistically relevant electronic and functional-group information that enhances the modeling of receptor-mediated and metabolism-related toxicity endpoints.

Collectively, these results demonstrate that: (1) all multimodal configurations outperform their unimodal counterparts; (2) the Graph–SMILES pair serves as the core predictive foundation; (3) Image and Spectrum modalities contribute complementary visual and electronic perspectives. Thus, the proposed attention-based fusion network effectively integrates heterogeneous sources of molecular information, yielding both higher accuracy and greater representational diversity.

### 5.3 Task-specific analysis

A detailed analysis of performance across the 12 toxicity endpoints in the Tox21 dataset reveals a sophisticated interplay between data modalities and toxicological mechanisms (Tables 2, 3). Single-modality models show distinct strengths, indicating that different molecular representations are better suited for capturing specific biological activities. For instance, the Graph encoder performed best on the NR-AR-LBD endpoint with an ROC-AUC of 0.874±0.059 . In contrast, the SMILES encoder was the top-performing single-modality model for the SR-p53 endpoint, with an ROC-AUC of 0.806±0.041 .

**TABLE 2:** Mean ( ± std) ROC-AUC on the 7 Nuclear Receptor (NR) endpoints.

Model (encoder)	NR-AR	NR-AR-LBD	NR-AhR	NR-Aro	NR-ER	NR-ER-LBD	NR-PPAR- γ
Graph	0.765±0.097	0.874±0.059	0.858±0.024	0.793±0.042	0.761±0.023	0.822±0.108	0.835±0.056
SMILES	0.780±0.089	0.855±0.089	0.851±0.031	0.784±0.038	0.773±0.056	0.865±0.053	0.796±0.082
Image	0.750±0.020	0.789±0.078	0.738±0.104	0.679±0.037	0.737±0.038	0.782±0.084	0.700±0.069
Spectrum	0.612±0.145	0.605±0.163	0.705±0.062	0.685±0.020	0.666±0.045	0.732±0.115	0.639±0.089
MoltiTox-3	0.783±0.076	0.864±0.064	0.877±0.024	0.801±0.038	0.780±0.046	0.863±0.054	0.824±0.044
MoltiTox	0.801±0.065	0.870±0.066	0.875±0.022	0.803±0.034	0.794±0.036	0.868±0.057	0.828±0.043

Results are averaged over five independent scaffold splits, with each value representing the test ROC-AUC.

Bold values indicate the highest test ROC-AUC across all models.

**TABLE 3:** Mean ( ± std) ROC-AUC on the 5 Stress Response (SR) endpoints.

Model	SR-ARE	SR-ATAD5	SR-HSE	SR-MMP	SR-p53
Graph	0.748 ± 0.020	0.834 ± 0.060	0.779 ± 0.033	0.843 ± 0.024	0.758 ± 0.019
SMILES	0.766 ± 0.035	0.818 ± 0.060	0.783 ± 0.061	0.864 ± 0.025	0.806±0.041
Image	0.680 ± 0.097	0.678 ± 0.086	0.715 ± 0.059	0.761 ± 0.060	0.681 ± 0.081
Spectrum	0.736 ± 0.045	0.729 ± 0.078	0.618 ± 0.110	0.724 ± 0.047	0.715 ± 0.081
MoltiTox-3	0.781 ± 0.036	0.845±0.064	0.799 ± 0.041	0.880±0.012	0.805±0.016
MoltiTox	0.791±0.024	0.856±0.053	0.806±0.036	0.875 ± 0.014	0.803 ± 0.016

Results are averaged over five independent scaffold splits, with each value representing the test ROC-AUC.

Bold values indicate the highest test ROC-AUC across all models.

The MoltiTox-3 model consistently acts as a generalist, delivering strong, balanced performance across nearly all endpoints. In many cases, it surpasses the best individual encoder for a given task. On the NR-AhR endpoint, for example, the three-modality model achieved an ROC-AUC of 0.877±0.024 , exceeding the Graph encoder’s score of 0.858±0.024 . This result demonstrates that attention-based fusion of graph, SMILES, and image representations yields a synergistic effect, producing a molecular embedding more powerful than the sum of its parts.

Adding ^13^C NMR spectra as a fourth modality further enhances performance across most toxicological endpoints. The inclusion of spectral data yields improvements across 9 of the 12 tasks compared to MoltiTox-3. Among the nuclear receptor tasks, the most substantial gains were observed for NR-AR ( 0.783±0.076 to 0.801±0.065 ) and NR-ER ( 0.780±0.046 to 0.794±0.036 ). For the stress response endpoints, marked enhancements were also observed for SR-ATAD5 ( 0.845±0.064 to 0.856±0.053 ) and SR-ARE ( 0.781±0.036 to 0.791±0.024 ).

A noteworthy benefit of incorporating spectral data is the reduction in prediction variance. For instance, Across the nuclear receptor tasks, NR-AR variance dropped from ±0.076 to ±0.065 , and SR-ARE from ±0.036 to ±0.024 . This enhanced stability suggests that the electronic environment information captured by ^13^C NMR spectra provides complementary signals that improve model robustness, indicating that spectral data offers both mechanistic insights and improved generalization across different data splits.

### 5.4 Comparison with benchmarking models

To evaluate MoltiTox against established methods, we benchmarked its performance on the Tox21 dataset (Table 4). All reported metrics correspond to the test ROC-AUC, averaged over five independent scaffold splits to ensure fair and reliable comparison. Our model achieves the best performance among all compared models with a mean ROC-AUC of 0.831±0.032 . It outperforms all single-modality baselines, including the strongest graph-based model, D-MPNN (0.806±0.013) , and the top-performing SMILES-based model, MolFormer-XL (0.811±0.041) . Such consistent improvements across modalities highlight the effectiveness of the proposed multimodal fusion approach.

**TABLE 4:** Comparison of mean ( ± std) ROC-AUC across various models on the Tox21 dataset.

Model	Modality	ROC-AUC
ImageMol (Zeng et al., 2022)	Image	0.735 ± 0.014
SVM (Cortes and Vapnik, 1995)	ECFP	0.743 ± 0.031
GROVER-large (Rong et al., 2020)	Graph	0.783 ± 0.016
MolCLR (Wang et al., 2022)	Graph	0.799 ± 0.030
D-MPNN (Yang et al., 2019)	Graph	0.806 ± 0.013
MolFormer-XL (Ross et al., 2022)	SMILES	0.811 ± 0.041
MoltiTox	Graph + SMILES + image + spectrum	0.831±0.023

Results are averaged over five independent scaffold splits, with each value representing the test ROC-AUC. For each model, the corresponding data modality used for prediction is also presented.

Bold values indicate the highest test ROC-AUC across all models.

The primary contribution of this work lies in introducing and validating an approach for integrating multiple molecular representations, including spectroscopic information. Our evaluation across five independent scaffold splits provides a realistic estimate of generalization performance. These findings position MoltiTox as a methodological advancement that offers a blueprint for more holistic models in computational toxicology.

### 5.5 Interpretability of attention-based fusion

**FIGURE 3:**
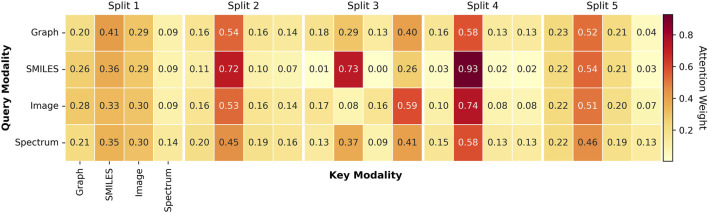
Attention weight heatmaps across modalities. Representative heatmaps visualize attention distributions learned by the multimodal fusion module across five scaffold splits. Rows correspond to the query modality, and columns correspond to the key modality. Each axis represents four input modalities: Molecular Graph, SMILES string, 2D molecular image, and ^13^C NMR spectrum, each processed by their respective encoders. Darker red cells indicate higher attention weights between modality pairs.

To further understand how MoltiTox integrates heterogeneous modalities, we analyzed the attention distributions learned by the fusion network. Representative attention heatmaps and averaged modality weight distributions are provided in Figure 3. Because the current attention module is task-agnostic and shared across all 12 endpoints, we summarized the results at the split level rather than at the endpoint level. Across five scaffold splits, SMILES embeddings received the highest average attention in three of the five runs, SMILES and Image were co-dominant in one, and Spectrum dominated in another. This pattern indicates that the model adaptively balances modality contributions depending on data composition.

The consistent prominence of SMILES suggests that sequence-based representations act as a stable “linguistic backbone,” providing a latent structure that aligns features from graph, image, and spectral encoders. Rather than reflecting overreliance, this behavior highlights the role of SMILES as a unifying representation that anchors other modalities and facilitates coherent multimodal integration. In contrast, the Spectrum modality generally received lower average attention across splits, yet in one of the five runs it dominated over other modalities.This variability suggests that spectral information can become more influential in subsets of data where electronic-environment cues are more informative for prediction. Thus, the Spectrum encoder likely contributes complementary chemical information related to atomic electronic environments and functional group characteristics, which may play a role in certain types of toxicity endpoints.

### 5.6 Chemical space visualization

To verify that the scaffold-based data partitioning preserved structural diversity and prevented bias in model evaluation, we analyzed the chemical space distribution of the five independent scaffold-based splits using t-distributed Stochastic Neighbor Embedding (t-SNE) (Maaten and Hinton, 2008) visualization. Each compound was first converted to its Bemis–Murcko scaffold and then encoded as a 2048-bit Morgan fingerprint (radius = 2) (Rogers and Hahn, 2010), capturing atomic neighborhoods within two bonds of each atom. These fingerprint vectors were projected onto a two-dimensional space using t-SNE with Euclidean distance, producing a unified coordinate system where each molecule occupied an identical position across all five splits.

**FIGURE 4:**
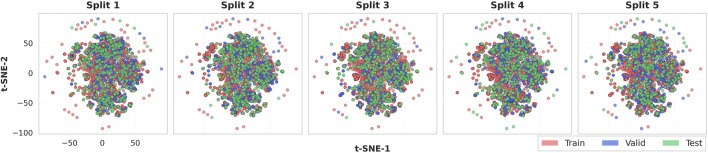
Unified t-SNE visualization of chemical space across five independent scaffold splits. All molecules are mapped to a shared two-dimensional coordinate system based on Morgan fingerprints derived from Bemis-Murcko scaffolds. Each subplot shows the train (red), validation (blue), and test (green) partitions for the corresponding split. The consistent positioning across splits demonstrates that the same molecules occupy identical coordinates, with only their train/valid/test assignments differing between splits.

Figure 4 illustrates the chemical space distribution across five independent scaffold splits. Each plot displays a consistent, densely populated central region with moderately scattered peripheral molecules, reflecting the overall scaffold diversity of the Tox21 dataset. Train (red), validation (blue), and test (green) compounds are well interspersed throughout both the central and peripheral regions, indicating that each partition covers a comparable range of structural motifs rather than being biased toward specific scaffold types. Although training compounds dominate numerically due to the 8:1:1 split ratio, validation and test molecules are evenly distributed across the chemical landscape. This consistent spatial pattern across all five splits confirms that scaffold-based partitioning maintains balanced structural diversity while preventing scaffold overlap between partitions, supporting the reliability of subsequent performance evaluation.

## 6 Discussion

The experimental results in Section 5 provide compelling evidence for the efficacy of a comprehensive multimodal approach in molecular toxicity prediction. The superior performance of MoltiTox validates the hypothesis that fusing diverse molecular representations can overcome the limitations of single-modality models. This section synthesizes these findings, discussing the implications for molecular machine learning, the unique value of spectroscopic data, and the limitations and potential directions for future work.

Our three-modality model, MoltiTox-3, achieved a mean ROC-AUC of 0.824, while the complete four-modality model, MoltiTox, reached a score of 0.831. Compared to the strongest single-modality baseline (SMILES, 0.811), these represent relative improvements of approximately 1.6% and 2.5%, respectively. These results confirm that fusing complementary information from heterogeneous sources yields a more comprehensive molecular embedding, thereby improving predictive accuracy and robustness.

The complementary contributions of each modality can be summarized as follows: the Graph modality captures molecular topology; the SMILES modality leverages sequential chemical language features (Ross et al., 2022); and the Image modality recognizes visual motifs (Zeng et al., 2022).

The inclusion of ^13^C NMR spectra offers complementary information about the electronic and local chemical environment that is not fully captured by topology-, sequence-, or image-based representations. In our experiments, the performance gains from adding NMR were generally modest but consistent, with the most notable improvements observed for NR-AR (0.783 → 0.801), NR-ER (0.780 → 0.794), and SR-ATAD5 (0.845 → 0.856), alongside reduced prediction variance across splits. Importantly, our goal is not merely to detect the presence of specific functional groups, but to exploit richer spectral signatures such as chemical-shift patterns that reflect neighboring-group effects and electronic environments. Taken together, these results indicate that NMR contributes selectively on endpoints where electronic context is particularly informative, while providing incremental benefits overall under a rigorous scaffold-split evaluation.

By leveraging the strengths of multiple modalities, MoltiTox not only achieves competitive performance but also provides balanced generalization across a range of endpoints. The success of MoltiTox offers several important implications for computational toxicology and molecular machine learning. First, it validates the superiority of a multimodal deep learning model for toxicity prediction. Traditional QSAR models often fail to generalize due to inconsistent data and an inability to account for metabolic processes (Cronin et al., 2025). Similarly, single-modality deep learning models are constrained by biases toward specific types of toxicological signals. Our results confirm that a fused representation generalizes more effectively across toxicity endpoints, delivering balanced and accurate performance. This suggests that future *in silico* toxicology should move beyond the ‘one molecule, one modality’ paradigm. In particular, the dramatic performance gain from ^13^ C NMR data underscores that spectra contain information that purely structure-based models can miss. Integrating such data could therefore strengthen biochemical activity prediction and be extended to other analytical domains, like mass spectrometry.

Furthermore, MoltiTox uses multi-task learning to predict 12 related endpoints simultaneously. This approach induces a beneficial inductive bias by sharing learned representations across tasks, which can act as an effective regularizer (Caruana, 1997). When latent molecular features are common to multiple endpoints, this method reinforces predictive signals, providing an effect analogous to data augmentation in data-scarce scenarios (Mayr et al., 2016). This multi-task setting creates indirect learning opportunities, allowing tasks with few positive examples to draw upon signals from data-rich tasks.

Beyond its methodological and predictive contributions, MoltiTox also has potential implications for regulatory toxicology. The proposed multimodal embeddings, which integrate structural, physicochemical, and spectroscopic information, could serve as a foundation for mechanistically grounded chemical similarity metrics. Such representations may support *read-across* approaches by identifying compounds that share both structural and electronic features relevant to toxicity. They could also enhance the reliability of *Threshold of Toxicological Concern* (TTC) evaluations by improving predictive accuracy for data-poor chemicals, and contribute to the validation of *New Approach Methodologies* (NAMs) by providing interpretable, mechanistically informed AI models. Furthermore, the MoltiTox framework could be aligned with ongoing initiatives such as Tox21 and ToxCast, enabling integration of multimodal AI-based predictions into established risk assessment pipelines and facilitating regulatory decision support.

Finally, we integrated embeddings using an attention-based fusion strategy. This design enables the model to modulate interactions between modalities through learnable, sample-specific attention weights. Unlike simple concatenation, a self-attention mechanism learns a fused representation that captures non-linear interactions across modalities. Our implementation adopts the multi-head attention mechanism from Vaswani et al. (2017), treating each modality embedding as a token and using multiple heads to attend to different signals. This process facilitates the interpretation of modality contributions, providing a promising path forward for multimodal toxicity prediction.

## 7 Limitations and future work

Despite its success, this study has several limitations that open avenues for future research. The primary constraint is the sparsity of spectral data. With ^13^ C NMR spectra available for only about 36% of the Tox21 compounds, MoltiTox and the spectrum encoder was trained on a relatively small subset of the dataset. This data scarcity likely prevented the encoder from reaching its full potential and contributed to the higher variance observed in the four-modality model. Future work could incorporate advances in spectral prediction to generate high-fidelity *in silico* spectra for compounds with missing data. This data augmentation strategy could unlock the full potential of the spectroscopic modality by enabling the encoder to train on the complete dataset.

Beyond data augmentation, future work will also extend this study toward mechanistic interpretation of NMR signals by mapping characteristic ^13^C shift regions (e.g., 110–150 ppm aromatic; 160–220 ppm carbonyl) to functional group reactivity and toxicity pathways. Planned analyses include region-level ablation and SHAP-based attribution to clarify how spectroscopic features relate to electrophilicity, metabolism, and receptor-specific endpoints.

A second limitation is the task-agnostic design of the fusion mechanism. The current multi-head self-attention module learns a single set of fusion weights that is applied uniformly across all 12 toxicity prediction tasks. However, our results clearly show that the importance of each modality is highly task-dependent. Future work should therefore explore task-conditioned fusion mechanisms. One solution is to introduce learnable task embeddings that allow the model to dynamically prioritize modalities for a specific endpoint. For example, such an architecture could learn to up-weight NMR data for the NR-PPAR- γ task while disregarding it for the SR-MMP task. This would mitigate task-specific performance degradation and improve overall accuracy.

Finally, the computational cost of a model with four large, independent encoders is considerable. While effective, this approach may limit practicality in some settings. Future research should investigate more computationally efficient architectures. Promising directions include using knowledge distillation to transfer knowledge to smaller student models or developing adaptive inference methods that selectively use only the most informative modalities for a given prediction.

In addition to the directions above, future work may also explore alternative molecular representations to further enhance robustness. For instance, SELFIES (Self-Referencing Embedded Strings) (Krenn et al., 2020) offers a chemically valid and surjective encoding that eliminates syntactic invalidity issues inherent to SMILES. Although SMILES was used in this study due to its wide adoption and compatibility with pre-trained language models such as MolFormer-XL, incorporating SELFIES-based tokenization in future Transformer architectures could improve the structural fidelity and consistency of learned molecular embeddings.

Furthermore, extending the evaluation to independent external toxicity datasets would strengthen the assessment of model generalizability. However, acquiring all four modalities, particularly ^13^C NMR spectra, for external datasets would require substantial additional curation effort due to limited coverage in public NMR repositories. We plan to address this limitation in future work once multimodal datasets with more comprehensive spectral coverage become available.

## 8 Conclusion

In this paper, we proposed MoltiTox, a multimodal model for molecular toxicity prediction. This work combines four heterogeneous molecular data modalities, graphs, SMILES sequences, 2D images, and ^13^C NMR spectra, through simultaneous fusion within a single architecture. We designed modality-specific encoders and an attention-based fusion mechanism to learn a composite representation capturing each compound’s multifaceted properties. Results on the Tox21 benchmark demonstrate that MoltiTox outperforms single-modality approaches, achieving a mean ROC-AUC of 0.831 across 12 toxicity endpoints. Beyond the aggregate result, the ^13^C NMR modality provided selective yet consistent benefits on endpoints where electronic context is particularly informative, with the largest gains observed for NR-AR, NR-ER, and SR-ATAD5, alongside reduced prediction variance across splits. These improvements likely arise because full spectra encode continuous, environment-sensitive cues (e.g., chemical-shift dispersion and deshielding patterns). While the average gain is modest, it remains consistent under a rigorous scaffold split and incurs minimal inference-time overhead. In conclusion, MoltiTox illustrates that a holistic, multimodal approach overcomes the limitations of single-modality models to achieve more accurate and robust toxicity predictions. This contribution will help prioritize compounds for testing, reduce costly late-stage failures, and ultimately advance *in silico* screening as a critical component of safer and more effective drug development.

## Data Availability

The Tox21 dataset used in this study is publicly available through MoleculeNet (Wu et al., 2018). The ^13^C NMR spectral data were obtained from publicly accessible spectral databases: NMRShiftDB (Steinbeck et al., 2003), NP-MRD (Wishart et al., 2022), and HMDB (Wishart et al., 2007). All preprocessing scripts and the processed dataset combining molecular structures and spectral vectors (including the 2,851-compound subset) have been deposited in a private GitHub repository (https://github.com/skku-aihclab/proj25-molecule-toxicity-prediction). The repository will be made publicly accessible upon publication of this article to ensure reproducibility and facilitate external benchmarking.
