# Supplementary material for: MoltiTox: a multimodal fusion model for molecular toxicity prediction
Source: Front Toxicol. 2025 Dec 18;7:1720651. doi: 10.3389/ftox.2025.1720651 (PMC12756560; doi:10.3389/ftox.2025.1720651)
Supplement: Supplementary file 1 [file DataSheet1.pdf]

Supplementary Material

1 SUPPLEMENTARY TABLES AND FIGURES

The representative hyperparameters reported in Tables S2 and S3, as well as the dataset splits summarized in Table S1, are taken from the run that achieved the median validation performance among five independent seeds.

We provide a brief description of the hyperparameters. Unless otherwise noted, `emb_dim` refers to the dimension of the final output embedding.

For the Single-modality encoders, `emb_dim` is the output embedding dimension; `lr` and `weight_decay` are the learning rate and weight decay, respectively. Specifically, for the Graph Encoder, `hidden_dim` refers to the hidden layer size in each GINEConv block and `num_layers` specifies the number of stacked GINEConv layers. For the Image Encoder, `hidden_size` indicates the hidden size of MLP layers following the CNN backbone. For the Spectrum Encoder, `hidden_dim` denotes the hidden layer size in the MLP projection head. For the Multimodal fusion models, `emb_dim` is the dimension to which each modality-specific embedding is projected before fusion; `hidden_dim` is the hidden size of the MLP layers in the fusion head; and `dropout` is the dropout rate applied to the fusion MLP layers.

Table S1. Dataset splits for all compounds and the ^{13}C NMR subset.

Dataset	Train	Valid	Test
All Compounds	6,257	783	783
Compounds with ^{13}C NMR spectrum	2,466	206	179

Table S2. Optimal hyperparameters for the four single-modality encoders. The values were determined using an Optuna search.

Parameter	Graph	SMILES	Image	Spectrum
`hidden_dim`	256	–	–	512
`hidden_size`	–	–	256	–
`num_layers`	5	–	–	–
`emb_dim`	512	768	256	256
`lr`	3×10^{-4}	3×10^{-5}	3×10^{-4}	1×10^{-3}
`weight_decay`	1×10^{-5}	1×10^{-5}	1×10^{-4}	1×10^{-4}

Table S3. Optimal hyperparameters for the MoltiTox fusion models.

Parameter	3-modality	4-modality
`emb_dim`	128	128
`hidden_dim`	512	512
`lr`	3×10^{-4}	3×10^{-4}
`weight_decay`	1×10^{-4}	1×10^{-4}
`dropout`	0.5	0.5

Tables S4–S6 present the full set of performance metrics for all 12 models across the Train, Validation, and Test splits of the Tox21 dataset. Across all metrics, multimodal fusion models consistently demonstrate improved generalization performance compared to single-modality baselines. While all models achieve high overall Accuracy and Specificity, single-modality encoders tend to exhibit lower Balanced Accuracy and Sensitivity, indicating limited recall for minority toxic classes. In contrast, multimodal architectures such as MoltiTox and MoltiTox-3 achieve higher Balanced Accuracy and Cohen's Kappa values, suggesting better class discrimination and robustness across endpoints. The Matthews Correlation Coefficient (MCC) further supports this observation, showing stronger correlation between predictions and ground truth labels for the multimodal models. Notably, the gap between training and validation performance remains moderate for the fusion networks, implying effective regularization and minimal overfitting despite their higher representational capacity. These supplementary results reinforce the main findings that integrating heterogeneous molecular representations improves not only ROC-AUC but also a wide range of complementary performance metrics.

Table S4. Performance metrics (Train split) for 12 models on the Tox21 dataset. Results are averaged over five independent scaffold splits, with each value representing the mean ± standard deviation. Metrics include Accuracy, Balanced Accuracy, Cohen's Kappa, Matthews Correlation Coefficient (MCC), Sensitivity, and Specificity.

Model	Accuracy	Balanced Acc.	Cohen's Kappa	MCC	Sensitivity	Specificity
Single-Modality Baselines						
Graph Encoder	0.951 ± 0.004	0.688 ± 0.025	0.488 ± 0.051	0.537 ± 0.042	0.383 ± 0.050	0.992 ± 0.002
SMILES Encoder	0.953 ± 0.012	0.688 ± 0.083	0.465 ± 0.187	0.508 ± 0.174	0.384 ± 0.166	0.992 ± 0.004
Image Encoder	0.931 ± 0.006	0.599 ± 0.046	0.241 ± 0.083	0.279 ± 0.077	0.215 ± 0.108	0.982 ± 0.018
Spectrum Encoder	0.947 ± 0.016	0.553 ± 0.110	0.131 ± 0.267	0.152 ± 0.282	0.106 ± 0.221	0.999 ± 0.001
Two-Modality Baselines						
Graph + SMILES	0.981 ± 0.004	0.882 ± 0.038	0.812 ± 0.062	0.819 ± 0.057	0.772 ± 0.076	0.993 ± 0.002
Graph + Image	0.962 ± 0.005	0.768 ± 0.028	0.636 ± 0.051	0.659 ± 0.044	0.545 ± 0.057	0.991 ± 0.002
Graph + Spectrum	0.953 ± 0.005	0.695 ± 0.035	0.496 ± 0.084	0.538 ± 0.080	0.399 ± 0.069	0.992 ± 0.001
SMILES + Image	0.983 ± 0.007	0.894 ± 0.067	0.830 ± 0.099	0.837 ± 0.088	0.795 ± 0.133	0.994 ± 0.001
SMILES + Spectrum	0.980 ± 0.010	0.874 ± 0.077	0.790 ± 0.141	0.799 ± 0.132	0.755 ± 0.152	0.993 ± 0.003
Image + Spectrum	0.946 ± 0.005	0.651 ± 0.034	0.388 ± 0.078	0.429 ± 0.072	0.311 ± 0.068	0.990 ± 0.002
Multimodal Models						
MoltiTox-3	0.981 ± 0.006	0.887 ± 0.047	0.821 ± 0.078	0.827 ± 0.070	0.782 ± 0.092	0.993 ± 0.003
MoltiTox	0.984 ± 0.004	0.911 ± 0.025	0.855 ± 0.040	0.858 ± 0.038	0.828 ± 0.049	0.993 ± 0.002

Table S5. Performance metrics (Validation split) for 12 models on the Tox21 dataset. Results are averaged over five independent scaffold splits, with each value representing the mean ± standard deviation. Metrics include Accuracy, Balanced Accuracy, Cohen's Kappa, Matthews Correlation Coefficient (MCC), Sensitivity, and Specificity.

Model	Accuracy	Balanced Acc.	Cohen's Kappa	MCC	Sensitivity	Specificity
Single-Modality Baselines						
Graph Encoder	0.914 ± 0.005	0.627 ± 0.022	0.315 ± 0.043	0.350 ± 0.042	0.275 ± 0.049	0.978 ± 0.006
SMILES Encoder	0.910 ± 0.004	0.620 ± 0.038	0.288 ± 0.089	0.315 ± 0.093	0.267 ± 0.082	0.972 ± 0.010
Image Encoder	0.895 ± 0.016	0.583 ± 0.040	0.190 ± 0.076	0.207 ± 0.073	0.204 ± 0.103	0.963 ± 0.027
Spectrum Encoder	0.897 ± 0.017	0.509 ± 0.015	0.023 ± 0.039	0.030 ± 0.045	0.024 ± 0.044	0.994 ± 0.014
Two-Modality Baselines						
Graph + SMILES	0.906 ± 0.007	0.643 ± 0.018	0.329 ± 0.039	0.347 ± 0.037	0.328 ± 0.041	0.959 ± 0.008
Graph + Image	0.911 ± 0.005	0.647 ± 0.020	0.350 ± 0.043	0.370 ± 0.042	0.324 ± 0.043	0.969 ± 0.005
Graph + Spectrum	0.914 ± 0.005	0.621 ± 0.014	0.294 ± 0.040	0.326 ± 0.050	0.263 ± 0.027	0.979 ± 0.003
SMILES + Image	0.909 ± 0.007	0.655 ± 0.032	0.357 ± 0.067	0.375 ± 0.063	0.350 ± 0.067	0.968 ± 0.017
SMILES + Spectrum	0.906 ± 0.005	0.646 ± 0.026	0.330 ± 0.065	0.343 ± 0.070	0.331 ± 0.053	0.959 ± 0.007
Image + Spectrum	0.903 ± 0.006	0.596 ± 0.024	0.230 ± 0.050	0.245 ± 0.050	0.221 ± 0.053	0.971 ± 0.005
Multimodal Models						
MoltiTox-3	0.904 ± 0.008	0.645 ± 0.023	0.333 ± 0.057	0.346 ± 0.056	0.332 ± 0.042	0.958 ± 0.007
MoltiTox	0.905 ± 0.005	0.656 ± 0.013	0.356 ± 0.042	0.368 ± 0.046	0.355 ± 0.023	0.958 ± 0.009

Table S6. Performance metrics (Test split) for 12 models on the Tox21 dataset. Results are averaged over five independent scaffold splits, with each value representing the mean ± standard deviation. Metrics include Accuracy, Balanced Accuracy, Cohen's Kappa, Matthews Correlation Coefficient (MCC), Sensitivity, and Specificity.

**Model**	**Accuracy**	**Balanced Acc.**	**Cohen's Kappa**	**MCC**	**Sensitivity**	**Specificity**
Single-Modality Baselines						
Graph Encoder	0.911 ± 0.008	0.628 ± 0.028	0.320 ± 0.063	0.359 ± 0.062	0.275 ± 0.060	0.980 ± 0.005
SMILES Encoder	0.906 ± 0.008	0.622 ± 0.049	0.290 ± 0.095	0.316 ± 0.082	0.271 ± 0.114	0.974 ± 0.016
Image Encoder	0.850 ± 0.112	0.549 ± 0.034	0.114 ± 0.075	0.138 ± 0.086	0.177 ± 0.116	0.920 ± 0.139
Spectrum Encoder	0.891 ± 0.020	0.511 ± 0.019	0.027 ± 0.045	0.035 ± 0.048	0.030 ± 0.053	0.992 ± 0.015
Two-Modality Baselines						
Graph + SMILES	0.909 ± 0.010	0.638 ± 0.027	0.330 ± 0.068	0.358 ± 0.066	0.304 ± 0.054	0.973 ± 0.006
Graph + Image	0.911 ± 0.012	0.653 ± 0.027	0.368 ± 0.054	0.395 ± 0.050	0.333 ± 0.061	0.972 ± 0.012
Graph + Spectrum	0.911 ± 0.010	0.631 ± 0.032	0.316 ± 0.062	0.348 ± 0.060	0.283 ± 0.070	0.979 ± 0.008
SMILES + Image	0.906 ± 0.010	0.638 ± 0.018	0.319 ± 0.036	0.336 ± 0.034	0.307 ± 0.040	0.969 ± 0.006
SMILES + Spectrum	0.904 ± 0.010	0.608 ± 0.028	0.256 ± 0.065	0.277 ± 0.062	0.242 ± 0.063	0.974 ± 0.009
Image + Spectrum	0.901 ± 0.011	0.593 ± 0.027	0.224 ± 0.059	0.245 ± 0.064	0.210 ± 0.060	0.976 ± 0.011
Multimodal Models						
MoltiTox-3	0.914 ± 0.007	0.650 ± 0.021	0.353 ± 0.042	0.379 ± 0.043	0.326 ± 0.042	0.973 ± 0.003
MoltiTox	0.911 ± 0.008	0.654 ± 0.030	0.355 ± 0.058	0.380 ± 0.053	0.339 ± 0.063	0.969 ± 0.007
